# Current and Binge Drinking Among High School Students — United States, 1991–2015

**DOI:** 10.15585/mmwr.mm6618a4

**Published:** 2017-05-12

**Authors:** Marissa B. Esser, Heather Clayton, Zewditu Demissie, Dafna Kanny, Robert D. Brewer

**Affiliations:** ^1^Division of Population Health, National Center for Chronic Disease Prevention and Health Promotion, CDC; ^2^Division of Adolescent and School Health, National Center for HIV/AIDS, Viral Hepatitis, STD and TB Prevention, CDC.

Excessive drinking accounted for approximately 4,300 deaths each year among persons aged <21 years during 2006–2010,* and underage drinking cost the United States $24.3 billion in 2010 (*1*). CDC analyzed data from the national Youth Risk Behavior Survey (YRBS) for the years 1991–2015 to examine trends in drinking by U.S. high school students, and from the 2015 YRBS to assess the usual source of alcohol consumed^†^ and binge drinking intensity (i.e., the average number of drinks consumed per binge drinking occasion).^§^ During 1991–2007, the prevalence of current drinking^¶^ among high school students declined significantly, from 50.8% (1991) to 44.7% (2007), and then significantly declined to 32.8% in 2015. The prevalence of binge drinking** increased from 31.3% in 1991 to 31.5% in 1999, and then significantly declined to 17.7% in 2015. Most high school students who drank were binge drinkers (57.8%), and 43.8% of binge drinkers consumed eight or more drinks in a row. Despite progress, current drinking and binge drinking are common among high school students, and many students who binge drink do so at high intensity (i.e., eight or more drinks in a row). Widespread use of evidence-based strategies for preventing excessive drinking (e.g., increasing alcohol taxes, regulating alcohol outlet density, and having commercial host liability laws) could help reduce underage drinking and related harms.^††^

The national YRBS is a cross-sectional, biennial school-based survey of 9th–12th grade students in U.S. public and private schools that monitors the prevalence of health risk behaviors. During 1991–2015, a three-stage cluster sample design was used to select nationally representative samples of students; sample sizes ranged from 10,904 to 16,410. During each cycle, students completed an anonymous, self-administered questionnaire. Response rates ranged from 60% to 71%. Data were weighted to account for oversampling of non-Hispanic black and Hispanic students and nonresponse, and to produce national estimates of health risk behaviors among U.S. high school students who attend public or private schools. Details of the YRBS methodology have been published previously.^§§^

Current drinking was defined as consuming one or more alcoholic drink on ≥1 days during the past 30 days. Binge drinking was defined as consuming five or more alcoholic drinks in a row on ≥1 days during the past 30 days. The usual source of alcohol and binge drinking intensity also were assessed. The prevalence of current drinking was calculated among students overall. The prevalence of binge drinking was calculated among students overall and current drinkers. The usual source of alcohol and binge drinking intensity were calculated among current drinkers only.

Data from 1991–2015 were used to examine trends in current drinking, binge drinking, and binge drinking among current drinkers, adjusted for sex, race/ethnicity, and grade. Trends were analyzed using logistic regression models and interaction terms. Time was modeled as a continuous variable^¶¶^ with linear and nonlinear (quadratic) components, which were considered significant at p-values <0.05. Joinpoint software*** was used, when significant quadratic trends were found, to determine the year in which the trend changed direction or leveled off. Percentage-point changes were calculated to compare the magnitude of trends, but differences between subgroups were not tested for significance. Data from 2015 were used to assess the prevalence of drinking patterns overall and by sociodemographic characteristics, using pairwise t-tests to assess differences by subgroup. Respondents who had missing information were excluded from analyses.^†††^ The sample sizes presented are unweighted and the percentages are weighted.

The overall prevalence of current drinking among U.S. high school students declined significantly from 50.8% in 1991 to 44.7% in 2007, then further declined to 32.8% in 2015 (Figure 1). Trend analysis indicated that the prevalence of binge drinking increased from 31.3% in 1991 to 31.5% in 1999, then declined significantly to 17.7% in 2015. From 1991 to 2015, the percentage-point decline in the prevalence of current and binge drinking was greater among male students (current drinking declined 20.5 percentage points, and binge drinking declined 17.9 percentage points) than female students (current drinking declined 15.3 percentage points, and binge drinking declined 9.1 percentage points).

**FIGURE 1:**
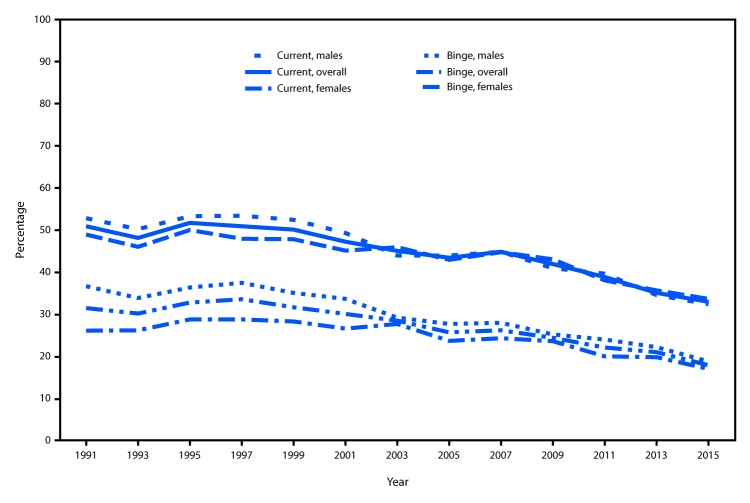
Prevalence of self-reported current drinking* and binge drinking**^†^** among high school students, by sex — Youth Risk Behavior Surveys, United States, 1991–2015 * One or more drinks of alcohol on ≥1 days during the 30 days before the survey. ^†^ Five or more drinks in a row (i.e., within a couple of hours) on ≥1 days during the 30 days before the survey.

The prevalence of binge drinking among current drinkers increased significantly from 62.2% in 1991 to 66.6% in 1997, then declined significantly to 57.8% in 2015 (Figure 2). Among male current drinkers, the prevalence of binge drinking declined significantly from 69.9% in 1991 to 61.5% in 2015. Among female current drinkers, the prevalence of binge drinking increased from 53.5% in 1991 to 60.4% in 1997, then declined to 54.0% in 2015.

**FIGURE 2:**
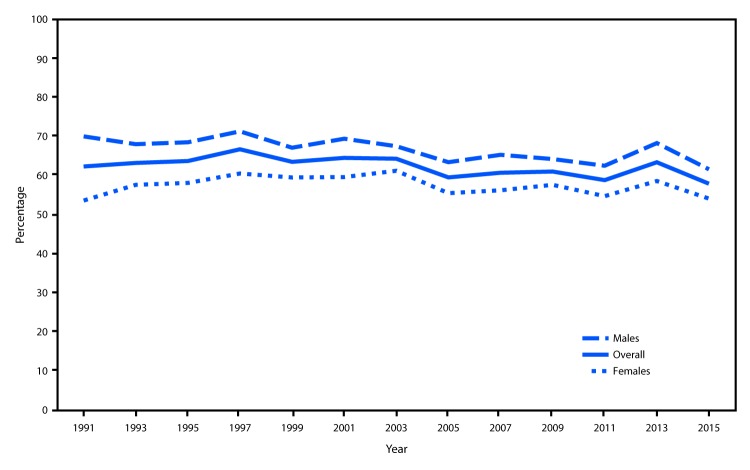
Prevalence of self-reported binge drinking* among high school students who reported current drinking,**^†^** by sex — Youth Risk Behavior Surveys, United States, 1991–2015 * Five or more drinks in a row (i.e., within a couple of hours) on ≥1 days during the 30 days before the survey. ^†^ One or more drinks of alcohol on ≥1 days during the 30 days before the survey.

In 2015, the prevalence of current drinking increased significantly with school grade from 23.4% among 9th grade students to 42.4% among 12th grade students, as did the prevalence of binge drinking, which was 10.4% among 9th grade students and 24.6% among 12th grade students (Table). Similarly, the prevalence of binge drinking among current drinkers increased significantly with school grade from 47.0% (9th grade students) to 61.9% (12th grade students). The prevalence of current and binge drinking was significantly higher among non-Hispanic white and Hispanic students than among non-Hispanic black students. The prevalence of binge drinking among current drinkers was also significantly higher among non-Hispanic white than among non-Hispanic black students.

**TABLE:** Weighted percentage of high school students who used alcohol, by selected characteristics — Youth Risk Behavior Survey, United States, 2015

Characteristic	All respondents (N = 15,624)		Current drinkers only (n = 4,659)
	Current drinking*	Binge drinking^†^	Binge drinking^†^
	Weighted % (95% CI)	Weighted % (95% CI)	Weighted % (95% CI)
**Overall**	**32.8 (30.4–35.2)**	**17.7 (15.8–19.8)**	**57.8 (54.6–60.9)**
**Sex**			
Male	32.2 (30.4–34.0)	18.6 (16.9–20.5)	61.5^§^ (57.4–65.4)
Female	33.5 (29.8–37.5)	16.8 (14.4–19.6)	54.0 (50.4–57.6)
**High school grade**			
9th	23.4 (20.9–26.1)^¶,^**	10.4 (9.1–11.8)^¶,^**^,††^	47.0 (40.6–53.6)^¶,^**^,††^
10th	29.0 (24.3–34.3)^¶,^**	15.1 (12.2–18.6)^ ¶,^**	56.5 (50.2–62.7)
11th	38.0 (34.6–41.4)**	22.1 (19.6–24.7)	61.4 (56.5–66.1)
12th	42.4 (38.4–46.4)	24.6 (21.5–28.0)	61.9 (57.8–65.9)
**Race/Ethnicity**			
White, non-Hispanic	35.2 (31.2–39.3)^§§^	19.7 (16.8–23.0)^§§^	59.5 (55.6–63.4)^§§^
Black, non-Hispanic	23.8 (18.6–30.0)^¶¶^	11.4 (8.8–14.7)^¶¶^	52.1 (47.0–57.2)
Hispanic	34.4 (31.9–37.0)	17.7 (15.8–19.7)	55.4 (51.6–59.2)

**Abbreviation:** CI = confidence interval.

* Had one or more drinks of alcohol on ≥1 days during the 30 days before the survey.

^†^ Had five or more drinks in a row (i.e., within a couple of hours) on ≥1 days during the 30 days before the survey.

^§^ Significantly different from female students.

^¶^ Significantly different from 11th grade students.

** Significantly different from 12th grade students.

^††^ Significantly different from 10th grade students.

^§§^ Significantly different from non-Hispanic black students.

^¶¶^ Significantly different from Hispanic students.

In 2015, 36.4% of binge drinkers and 55.7% of current drinkers who did not binge drink usually obtained alcohol from someone who gave it to them. Binge drinkers were more than three times more likely than current drinkers who did not binge drink to give someone money to purchase alcohol (30.7% compared with 8.8%) and to purchase alcohol themselves (8.8% compared with 2.8%). Among binge drinkers, 43.8% consumed eight or more drinks in a row. Among binge drinkers, the prevalence of consuming eight or more drinks in a row was significantly higher among male (50.5%) than female (35.3%) students.

## Discussion

Overall, current and binge drinking declined significantly among U.S. high school students from 1991 to 2015.^§§§^ The percentage-point decrease was greater among male than female students, and the prevalence of current and binge drinking among male and female students converged in recent years. However, approximately one in three high school students still drank alcohol and one in six were binge drinkers in 2015. Most high school students who drank were also binge drinkers, and in 2015, more than two in five binge drinkers consumed eight or more drinks in a row, increasing the risk for alcohol-attributable harms (e.g., violence, unintentional injuries, and alcohol poisoning). High school students who drank usually obtained alcohol from others, but binge drinkers were three times more likely than current drinkers who did not binge drink to give others money to purchase alcohol for them.

Other national surveys have also reported declines in current and binge drinking among high school–aged students since the 1990s, although specific prevalence estimates vary (*2*,*3*). The decline in underage drinking might be related to increased implementation of state underage drinking policies (*4*). Previous studies have also shown that the age 21 minimum legal drinking age was associated with reduced youth drinking (*5*) and reduced alcohol-attributable harms (e.g., motor vehicle crashes).^¶¶¶^ However, enforcement of this law varies across jurisdictions (*6*).

The finding that high school students who drink usually obtain alcohol from others, potentially including parents and guardians, is consistent with the state-specific relationship between youth and adult drinking (*7*). Policies affecting adults’ alcohol consumption have also been shown to reduce youth alcohol consumption significantly, and alcohol policies affecting the price and availability of alcohol consumption have been found to have the greatest impact on binge drinking by adults (*8*).

The findings in this report are subject to at least five limitations. First, YRBS data were only collected among teens who attended school, and therefore are not representative of all teens. Nationwide, in 2012, approximately 3% of persons aged 16–17 years were not enrolled in high school and had not completed high school.**** Second, YRBS data are self-reported, and alcohol consumption might not be accurately reported because of recall and social desirability biases. Third, the 1991–2015 YRBS period defines binge drinking for males and females as five or more drinks in a row, and the prevalence of binge drinking among females would likely be higher if it were assessed using a sex-specific, four-drink threshold (*9*). Fourth, data were not available to assess drinking by other racial/ethnic populations. Finally, it was not possible to evaluate reasons for the observed declines in current and binge drinking using YRBS data.

The Community Preventive Services Task Force recommends evidence-based strategies for reducing excessive alcohol use, including underage and binge drinking. These include increasing alcohol taxes, regulating alcohol outlet density, and having commercial host liability laws. Moreover, given the association between youth exposure to alcohol advertising and underage drinking, monitoring and reducing youth exposure to alcohol advertising through the implementation of “no-buy” lists (i.e., lists of television programming that risk overexposing youth to alcohol advertising based on the industry’s self-regulatory alcohol marketing guidelines) might also help reduce underage drinking (*10*).

**Summary**

**What is already known about this topic?**

Each year from 2006 to 2010, excessive alcohol consumption was responsible for approximately 4,300 deaths among persons aged <21 years, and, in 2010, underage drinking cost the United States $24.3 billion.

**What is added by this report?**

The overall prevalence of current drinking among U.S. high school students declined significantly from 50.8% in 1991 to 44.7% in 2007, then further declined to 32.8% in 2015. The prevalence of binge drinking increased from 31.3% in 1991 to 31.5% in 1999, then declined significantly to 17.7% in 2015. However, in 2015, approximately one in three high school students drank alcohol during the past 30 days and one in six were binge drinkers. Most high school students who drank (57.8%) were also binge drinkers, and more than two in five binge drinkers consumed eight or more drinks in a row.

**What are the implications for public health practice?**

Despite progress, current and binge drinking remain common among high school students, and many students who binge drink do so at high intensity (i.e., eight or more drinks in a row). Widespread use of evidence-based prevention strategies for excessive drinking (e.g., increasing alcohol taxes, regulating alcohol outlet density, and having commercial host liability laws) could help reduce underage drinking and related harms.

## Footnotes

*
https://www.cdc.gov/ARDI .

† Determined by responses to the question, “During the past 30 days, how did you usually get the alcohol you drank?”

§ Determined by responses to the question, “During the past 30 days, what is the largest number of alcoholic drinks you had in a row, that is, within a couple of hours?”

¶ Defined as reporting the consumption of one or more drinks of alcohol on ≥1 days during the 30 days before the survey.

** Defined as reporting the consumption of five or more drinks in a row (i.e., within a couple of hours) on ≥1 days during the 30 days before the survey.

††
https://www.thecommunityguide.org/sites/default/files/assets/What-Works-Alcohol-factsheet-and-insert.pdf .

§§
https://www.cdc.gov/mmwr/preview/mmwrhtml/rr6201a1.htm .

¶¶ For time modeled as a continuous variable, CDC used orthogonal coefficients reflecting the biennial spacing of the surveys.

***
https://surveillance.cancer.gov/joinpoint/ .

††† The percentage missing ranged from 0.8% (sex) to 2.3% (race/ethnicity). Among all students, the percentage missing for current drinking was 9.7%, and for binge drinking was 4.2%.

§§§ The minimum legal drinking age of 21 years was implemented by all states by 1988.

¶¶¶ https://www.thecommunityguide.org/content/motor-vehicle-injury-alcohol-impaired-driving-maintaining-current-minimum-legal-drinking-age.

****
https://nces.ed.gov/pubs2015/2015015.pdf .
